# Minimizing Freshwater Consumption in the Wash-Off Step in Textile Reactive Dyeing by Catalytic Ozonation with Carbon Aerogel Hosted Bimetallic Catalyst

**DOI:** 10.3390/polym10020193

**Published:** 2018-02-15

**Authors:** Enling Hu, Songmin Shang, Xiaoming Tao, Shouxiang Jiang, Ka-Lok Chiu

**Affiliations:** Institute of Textiles and Clothing, The Hong Kong Polytechnic University, Hong Kong, China; enling.allen.hu@connect.polyu.hk (E.H.); xiao-ming.tao@polyu.edu.hk (X.T.); kinor.j@polyu.edu.hk (S.J.); ka-lok.chiu@polyu.edu.hk (K.-L.C.)

**Keywords:** minimizing water consumption, wash-off, waste effluent recycling, catalytic ozonation, chemical oxidation demand, effluent color, fabric color quality, reactive dyeing

## Abstract

In textile reactive dyeing, dyed fabrics have to be rinsed in the wash-off step several times to improve colorfastness. Thus, the multiple rinsing processes drastically increase the freshwater consumption and meanwhile generate massive waste rinsing effluents. This paper addresses an innovative alternative to recycle the waste effluents to minimize freshwater consumption in the wash-off step. Accordingly, catalytic ozonation with a highly effective catalyst has been applied to remedy the waste rinsing effluents for recycling. The carbon aerogel (CA) hosted bimetallic hybrid material (Ag–Fe_2_O_3_@CA) was fabricated and used as the catalyst in the degradation of residual dyes in the waste rinsing effluents by ozonation treatments. The results indicate the participation of Ag–Fe_2_O_3_@CA had strikingly enhanced the removal percentage of chemical oxidation demand by 30%. In addition, it has been validated that waste effluents had been successfully reclaimed after catalytic ozonation with Ag–Fe_2_O_3_@CA. They could be additionally reused to reduce freshwater consumption in the wash-off step, but without sacrificing the color quality of corresponding fabrics in terms of color difference and colorfastness. This study may be the first to report the feasibility of catalytic ozonation in minimization of freshwater consumption in the wash-off step in textile reactive dyeing.

## 1. Introduction

High dependence on freshwater is an outstanding problem in textile manufacturing for the production of value-added final textile products [1]. The textile industry generally consumes massive amounts of freshwater for processing raw textile substrates in wet conditions, such as dyeing, printing and finishing. At the same time, significant waste effluents are also generated. No matter which wet processes are applied, they cannot make full use of dyes and/or auxiliaries. Thus, some of these chemicals would remain in water baths and have to be disposed with the hosting baths, generating the highly-polluted wastewater. As a result, textile dyeing houses have been thought to be the most polluting unit among all industries, if considering both the volume and pollution load of effluents discharged [2].

By overviewing textile production processes, reactive dyeing for cellulosic substrates has been regarded as the most critical step contributing to the massive consumption of freshwater and also the discharging of waste color effluents. Although reactive dyes have been developed drastically to fulfill requirements from different perspectives, the shortcoming of the low fixation rate onto fabrics has not yet been fully resolved [3]. It was estimated that the effective utilization of reactive dyes could be as low as 40% of the initial dosage [4], which suggested that the remaining 60% of dyes may be present on fabrics by physical adsorption or in the dyeing bath in dissolved ionic forms. Regarding the residual dyeing bath containing most of dissolved ionic dyes, it will be disposed after appropriate treatments to reduce its adverse impact into the environment [5,6]. For those unfixed dyes attached onto the fabrics, multiple rinsing processes are essentially required to strip the loose dyes from the wet fabrics. Otherwise, these dyes will seriously decrease the colorfastness of the final product, which is highly likely to be rejected by the market consumers. The extensive rinsing processes inevitably lead to a high consumption of freshwater, and meanwhile increases the volume of wastewater discharged. Thus, minimization of water use is an urgent consideration in textile industry [7].

Currently, environmental regulations are growing more restricted than ever before. For example, in Mainland China, the General Office of the State Council issued a regulation entitled “Implementation Plan for Issuance of Disposal Permission for Polluting Wastes” (Index no.: 000014349/2016-00225) in November 2016; soon after, the Ministry of Environmental Protection issued a regulation entitled “Interim Regulation for Issuance of Disposal Permission for Polluting Wastes” (Index no.: 000014672/2017-00003) in January 2017. These actions suggest that more severe control of wastewater management is being enforced. For sustainable development in the textile industry, it is more urgent to update textile manufacturing processes than ever before. Thus, developing cleaner production processes for textiles is becoming an immediate need for sustainable consideration [8]. With this intention, advanced treatment technologies towards recycling of the grey/waste water have been considered, since they are more promising in the sustainable development of the textile industry. The application of H_2_O_2_ oxidation [9], UV photolysis [10], persulfate oxidation [11], gamma radiation [12], adsorption [13], ozonation [14], and many comprehensive techniques [4,15] were reported previously, showing the feasibility of reclaiming waste textile effluents for successive reuse; whereby ozonation, which uses ozone as oxidant, has been believed to be a promising technology due to the numerous advantages of ozone such as its high oxidation potential and easy application [16]. Especially when highly effective and active catalysts are applied, the merit of ozonation technology can be fully displayed [17]. Thereby, following two pioneer studies applying ozonation treatment for reuse of dyeing effluents [18,19], in one of our previous studies, we successfully enabled the regeneration of highly contaminative dyeing baths (known as the first spent baths or fixation baths in one-bath exhaustion dyeing) by catalytic ozonation for its reuse in successive dyeing [14].

However, the above catalytic ozonation technique predominantly focused on the reuse of highly polluting effluents to replace freshwater in dye exhaustion only. It cannot be directly used in the wash-off step. In general, an entire batch-wise dyeing cycle can be roughly divided into dye exhaustion, dye fixation, and wash-off, in sequence. Fabrics after dye fixation must be rinsed with multiple freshwater baths to strip unfixed dyes to secure the satisfactory colorfastness [20]. The regenerated waste dyeing effluents are useless in the wash-off step, as the high load of electrolytes presented has the reverse effect on the stripping of loose dyes from fabrics. This is because the massive amount of electrolytes which are added in the dye exhaustion bath on purpose in the beginning will enhance the substantivity of dyes approaching fabrics in a water bath, leading to them rarely stripping loose color from fabrics. As a consequence, the wash-off step generally prefers a water bath with few electrolytes. If considering water consumption only, the wash-off step tends to be more contaminative than the dye exhaustion and fixation step, as it demands more freshwater and generates more wastewater.

Since water saving is turning out to be the new trend in textile wet processing [21], a few efforts have been made to improve efficiency in the wash-off step, in terms of reducing rinsing time and water consumption. The addition of microsphere absorbent [22] and dye transfer inhibiting (DTI) polymers [23] in the wash-off bath were reported recently. The results indicated the number of rinsing processes and the total water consumption had been substantially reduced. However, it should be pointed out that the addition of extra chemicals may potentially increase the overall pollution load in the waste rinsing effluents, even though these attempts were applicable and effective in reducing water consumption. Considering this predicament, in order to minimize water consumption in the wash-off step and meanwhile decrease the overall pollution load in effluents, the ozonation treatment has been proposed in the present study. Unlike chemical dosing, the ozonation technique would reduce pollution load in effluents rather than bring additional organic contaminants. In the present study, the first rinsing bath was collected in the wash-off step and then reclaimed by catalytic ozonation to degrade residual dyes in the effluent. To facilitate dye degradation in the waste effluent, a hosted bimetallic hybrid catalyst was fabricated. Due to an excellent three-dimensional continuous porous structure, carbon aerogel (CA) has been proved to play important roles in catalysis in plenty of studies [24,25]. Thus, in the present study, CA was adopted as the substrate to host the bimetallic hybrid, silver/iron oxide (Ag–Fe_2_O_3_). The bimetallic hybrid has been applied in the current study for several reasons: (1) Ag–Fe_2_O_3_ possesses multivalence states which would be beneficial in catalytic ozonation [26]; (2) Ag–Fe_2_O_3_ has been proved to be highly effective in both catalytic oxidation and reduction [27,28]; (3) the CA hosted Ag–Fe_2_O_3_ material (Ag–Fe_2_O_3_@CA) can be facially prepared via a hydrothermal approach under relatively low temperature. It is expected that Ag–Fe_2_O_3_@CA could tangibly promote catalytic degradation of organic dyes in effluents.

**Figure 1:**
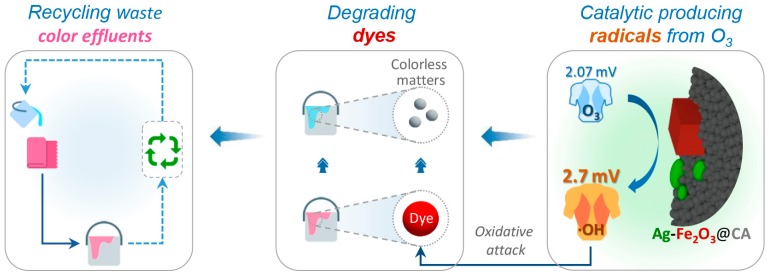
Ag–Fe_2_O_3_@CA facilitated catalytic ozonation for the recycling of waste rinsing effluents.

Based on this context, the present study primarily aims at evaluating the reusability of the first rinsing effluents. After treatment by catalytic ozonation, it should have equivalent performance to that of freshwater in stripping of loose dyes. The schematic of recycling waste effluents by catalytic ozonation with Ag–Fe_2_O_3_@CA can be seen in Figure 1. To begin with, freshwater was used for rinsing fabrics after dye fixation, and the resulted color effluent was collected instead of disposed of. In order to allow recycling of the waste effluent, the collected color effluent was afterwards reclaimed by catalytic ozonation. Due to the presence of Ag–Fe_2_O_3_@CA in catalytic ozonation, molecular ozone (O_3_) is anticipated to catalytically decompose into hydroxyl radicals (·OH), which have an even higher oxidation capacity and are thereby more capable to mineralize organics than O_3_ itself. Thus, during catalytic ozonation treatment, there are at least two available species which are responsible for dye degradation. As a result, residual dyes in color effluent can be degraded to colorless matters, including by-products from dye degradation, H_2_O and CO_2_, by oxidative attack from both O_3_ and ·OH. Because of the removal of organic pollutants in color effluents, the reclaimed effluents could be reused again in the subsequent rinsing processes, which would substantially minimize consumption of freshwater in the wash-off step. To examine the reusability of the reclaimed effluent, the color quality of the corresponding fabrics which were rinsed with reclaimed effluents will be evaluated in terms of color difference and fastness. The feasibility of recycling waste effluents by catalytic ozonation can be confirmed if the color quality of the fabrics which were rinsed with reclaimed effluent is not sacrificed compared to that of fabrics from conventional processes using freshwater.

Reviewing of the literature, there is very limited available information on reducing water use in the wash-off step in textile reactive dyeing. The present research is the first attempt to recycle waste rinsing effluents by catalytic ozonation for minimization of water consumption. This work is supposed to bring new insight in reducing the dependence on freshwater in textile reactive dyeing, and meanwhile extends the application of catalytic ozonation in the reclamation and recycling of textile wastewater.

## 2. Experimental

### 2.1. Materials and Chemicals

Semi-bleached cotton fabrics (250 g/m^2^) were purchased from Seven Seas Knitting & Dyeing Woks Ltd., Foshan, China. Before dyeing, the fabric was firstly pre-treated in a hot bath (0.1 g/L sodium dodecyl sulfate and 1.0 g/L Na_2_CO_3_) at 95 °C for 30 min, and then cleaned in warm baths thoroughly before drying in the air. The commercial reactive dye was used as received (Novacron Cheery S-D, Huntsman Corporation, Shanghai, China). Other chemicals used are all commercially available and of analytical grades.

### 2.2. Preparation of the Catalyst Material

The hosting material CA was prepared in our own laboratory [17]. Ag–Fe_2_O_3_@CA was prepared via hydrothermal approach. At the beginning, 10 mmol Fe(NO_3_)_3_ and 10 mmol AgNO_3_ were dissolved in 40 mL D.I. water, and then the solution was treated by ultrasonic for 30 min. Afterwards, 1 g of CA (20–30 mesh) was added into the above solution. For thorough impregnation of CA with Ag^+^ and Fe^3+^, the mixture was further treated by ultrasonic for an additional 3 h at room temperature. Subsequently 1 M NaOH solution was added dropwise into the above brown mixture under vigorous stirring, until the mixture obtained pH 10–11. After the addition of NaOH, the mixture was kept stirring for another 1 h. Later on, it was transferred into a 100 mL Teflon-lined stainless autoclave, which was then heated at 180 °C for 24 h. Towards the end, the resulting 20–30 mesh granular particles were rinsed with D.I. water and absolute ethanol 3 times repeatedly. The final catalyst was obtained by drying of the particles at 60 °C for 24 h in a vacuum oven.

### 2.3. Experimental Set-Up and Procedure

#### 2.3.1. Dyeing Procedure

**Figure 2:**
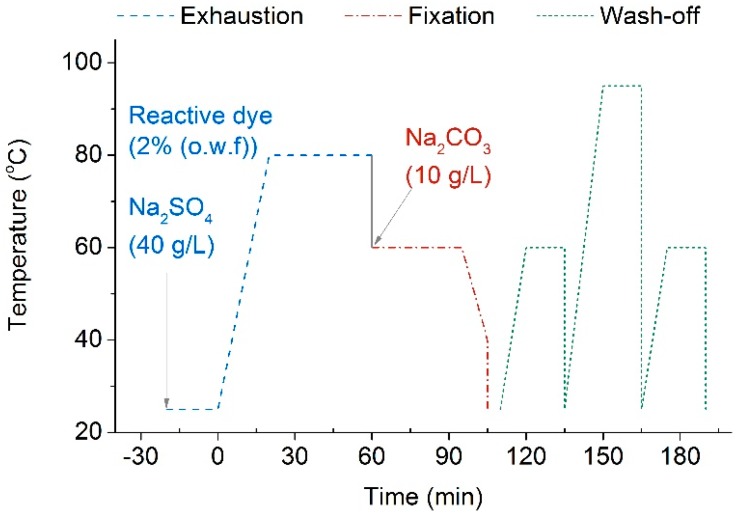
The entire dyeing curve for cotton knitting fabrics.

Dye exhaustion, dye fixation and wash-off experiments were all performed in the same dyeing machine (AHIBA IR™, Datacolor, Lawrenceville, NJ, USA). Firstly, naturally dried cotton fabrics (5 g/piece) after pre-treatment were separately soaked into 100 mL of dyeing baths, which contained 2% (o.w.f) reactive dye and 40 g/L Na_2_SO_4_. Then, the dyeing vessels were heated at 80 °C for 30 min for dye exhaustion. Soon after, fabrics were taken out of dyeing baths, which carefully added with Na_2_CO_3_ (10 g/L) in the absence of the fabrics. Afterwards, fabrics were re-immersed into dyeing baths containing Na_2_CO_3_, and kept at 60 °C for another 30 min for dye fixation. Eventually, fabrics were transferred from the water baths and padded by a horizontal padder to maintain 60% wet pick-up prior to the wash-off step. The entire dyeing curve can be found in Figure 2.

#### 2.3.2. Wash-Off Procedure

**Figure 3:**
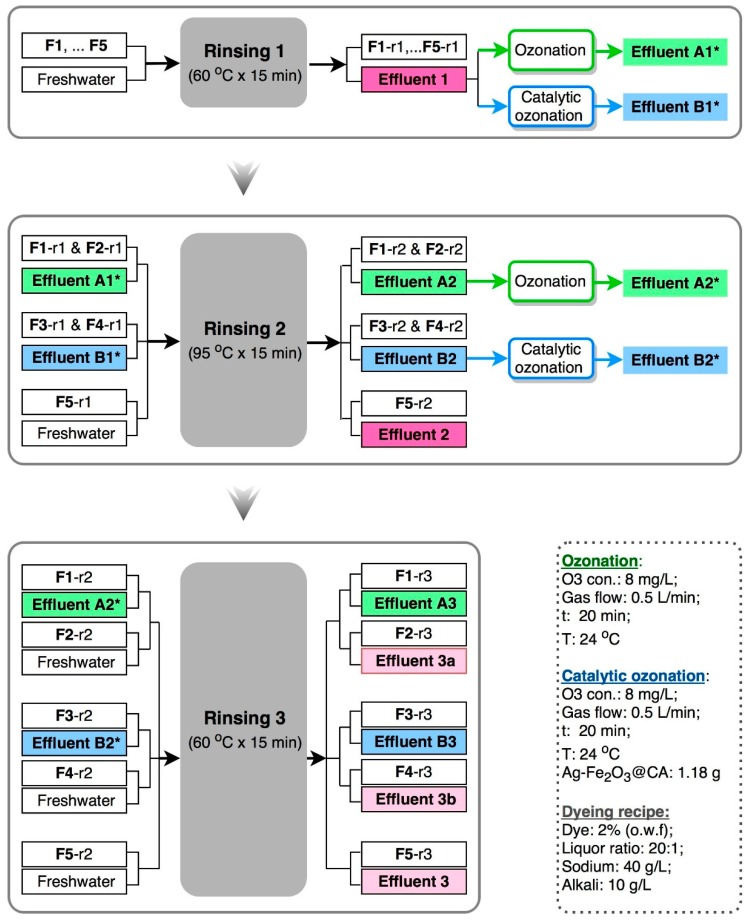
Flow chart for rinsing of fabrics in the wash-off step.

The flow chart of rinsing processes in the wash-off step is illustrated in Figure 3. Rinsing essays were performed 3 times for 5 groups of fabrics (F1, F2, F3, F4, F5). Each group has different treatment procedures, wherein F5 was selected as the reference group and was rinsed with freshwater in all of the rinsing processes. In Rinsing 1, all of the fabrics were cleaned with freshwater at 60 °C for 15 min, obtaining fabric sample F1-r1, F2-r1, F3-r1 and F4-r1. The related color effluents collected were then treated by ozonation alone and catalytic ozonation with Ag–Fe_2_O_3_@CA, resulting in Effluent A1* and Effluent B1*. Subsequently in Rinsing 2, Effluent A1* was used to clean F1-r1 and F2-r1 at 95 °C for 15 min to obtain fabric sample F1-r2 and F2-r2, and the resulted Effluent A2 was thereafter treated by ozonation alone to obtain Effluent A2*; likewise, Effluent B1* was used for cleaning of F3-r1 and F4-r1 to obtain fabric sample F3-r2 and F4-r2, and the resulted Effluent B2 was converted to Effluent B2* at the end by catalytic ozonation with Ag–Fe_2_O_3_@CA.

In Rinsing 3, Effluent A2* and Effluent B2* were collected for the final cleaning of F1-r2 and F3-r2 respectively; whereas freshwater was adopted for the cleaning of F2-r2 and F4-r2 for comparison. In the rinsing processes, some effluent samples were chosen for determination of color and chemical oxygen demand (COD), and the color quality of fabric samples F1-r3, F2-r3, F3-r3 and F4-r3 were compared to that of fabric sample F5-r3 after completion of the wash-off step.

#### 2.3.3. Reclamation of Dyeing Effluents by the Ozonation Treatment

Before the ozonation treatment, effluents collected were filtered through a filtration paper (0.45 µm) to remove suspended solid impurities. Ozonation was performed in a 0.5 L glass reactor. At the first beginning, CA (1 g) or Ag–Fe_2_O_3_@CA (1.18 g) may be dosed into the reactor which was pre-filled with 0.5 L of the waste rinsing effluent. The ozone gas, which was produced by the ozone generator (BM-02, Medozons, Nizhny Novgorod, Russia), continuously bubbled the effluents through a fine gas diffuser at the bottom of the reactor. After a certain time, 3 mL of the effluent sample was carefully transferred out. In order to remove trace solid catalysts which may impact COD determination, the effluent sample was pre-filtered slowly by the membrane filter (0.45 µm, Millipore, Burlington, MA, USA) prior to any testing.

### 2.4. Analytical Method

The structure and morphology of catalyst materials were investigated by X-ray diffraction (XRD) (SmartLab, Rigaku, Tokyo, Japan), Transmission electron microscope (TEM) (JEM-2100F, JEOL, Tokyo, Japan), and Scanning Electron Microscope (SEM) (VEGA3, Tescan, Brno-Kohoutovice, Czech Republic) with energy-dispersive X-ray spectroscopy (EDX). The N_2_ adsorption and desorption performance of catalyst materials were performed at 77 K on the surface area and pore size analyzer (Nova Touch LX^4^, Quantachrome Instruments, Boynton Beach, FL, USA). Thermal gravimetry analysis (TGA) (TGA/DSC 1 Star System, Mettler Toledo, Columbus, OH, USA) was carried out in air gas flow. Effluent color was directly measured by the absorbance (*A*) at the maximum absorbance wavelength (λ_max_ = 513 nm) on the UV-vis spectrophotometer (Lambda 18, Perkin-Elmer, Akron, OH, USA), or converted using Equation (1):

*Color* = *A_t_*/*A*_0_ (1)

where in *A*_0_ is the maximum initial absorbance of the dyeing effluent before ozonation and *A_t_* is the maximum absorbance of the dyeing effluent after ozonation for a specific time *t*. COD was evaluated according to the Hach Method 8000: oxygen demand, chemical. Each independent test was done triply and the average value was adopted for the sake of accuracy. For color quality determination, the CIELAB color space system was adopted to confirm the color specifications. The lightness (L*), chromaticity (a* and b*) and color difference (*ΔE_cmc(2:1)_*) of fabric samples were obtained by the spectrophotometer (Spectrum 650, Datacolor, Lawrenceville, NJ, USA) according to AATCC Test Method 173 [29]. In addition, colorfastness to laundering and crocking were evaluated following AATCC Test Method 61 and AATCC Test Method 8, respectively.

## 3. Results and Discussion

### 3.1. Characterization of Catalyst Materials

**Figure 4:**
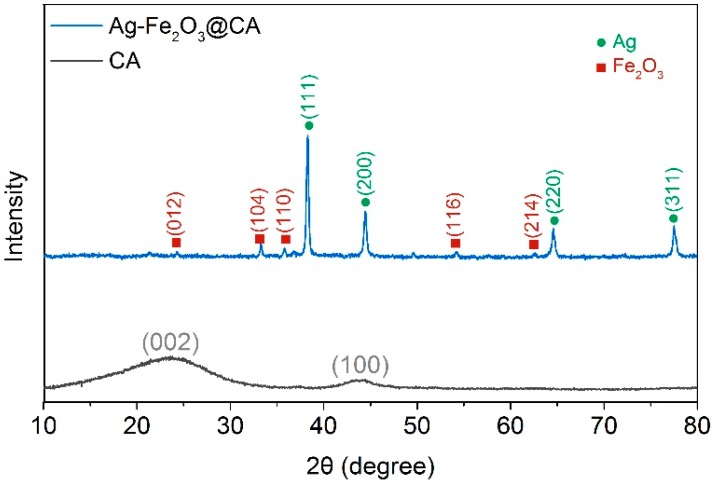
XRD pattern of carbon aerogel (CA) and Ag–Fe_2_O_3_@CA.

XRD patterns of Ag–Fe_2_O_3_@CA and pure CA are presented in Figure 4. For pure CA, the broad peaks located at 23° and 43°, which can be found in numerous carbon materials, were associated to the (002) and (100) planes of amorphous carbon and [30]. For Ag–Fe_2_O_3_@CA, the characteristic peaks at around 38°, 45°, 65° and 78° well matched up to the (111), (200), (221) and (311) planes of the face-centered cubic Ag in sequence according to the JCPDS File 04-0783 [31]. In addition, the (012), (104), (110), (116) and (214) lattice planes, which were observed at around 24°, 33°, 36°, 54° and 63° respectively, could be ascribed to the diffraction from α-Fe_2_O_3_ according to ICDD #01–079–0007 [32]. Last but not least, the diffraction peaks of Ag–Fe_2_O_3_@CA were all narrow and sharp, confirming that both Ag and α-Fe_2_O_3_ were of high crystallization.

**Figure 5:**
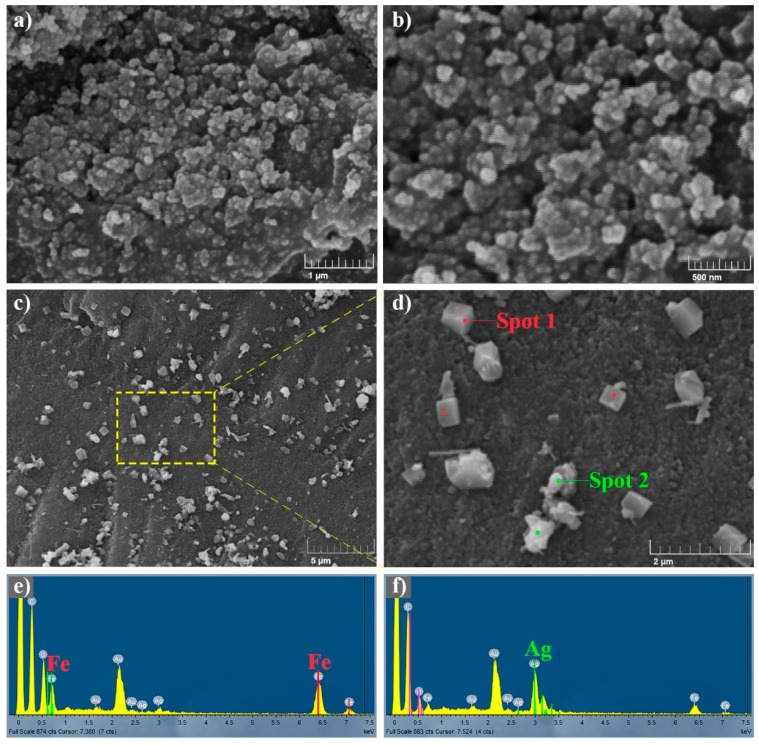
SEM images of CA (**a**,**b**) and Ag–Fe_2_O_3_@CA (**c**,**d**), and SEM-EDX spectra of Ag–Fe_2_O_3_@CA (**e**,**f**).

Figure 5 shows the SEM images of CA and Ag–Fe_2_O_3_@CA. As can be observed in Figure 5a,b, carbon particles agglomerated to form the CA substrate, which had a rough surface. Figure 5c shows the distribution of Ag–Fe_2_O_3_ nanoparticles over CA substrate. Particles were found clearly decorated on the surface of CA without obvious aggregation. For a closer observation, the selected region (yellow frame) in Figure 5c is magnified in Figure 5d. There were mainly two shapes of nanoparticles distributed on CA, the cubic (marked with red spots) and quasi-oval (marked with green spots) shapes. In order to identify these two nanoparticles, elemental analysis in SEM was performed by EDX to measure the intensity and energy distribution of the signals generated by the focused electron beam on the specific particles. EDX spectra of two typical spots (Spot 1 and Spot 2 in Figure 5d) were illustrated in Figure 5e,f respectively, and the related peaks were indexed by element Fe and Ag accordingly. According to elemental analysis, the cubic crystals could be assigned to Fe_2_O_3_, as the relative intensity of the typical Fe peaks in Figure 5e were remarkable than those from Ag. Likewise, the other quasi-oval nanoparticles can be identified as Ag nanocrystals, as Ag was more abundant than Fe according to Figure 5f. Besides this, the morphology of Spot 2 is also in line with that of Ag nanoparticles from a previous study [32].

**Figure 6:**
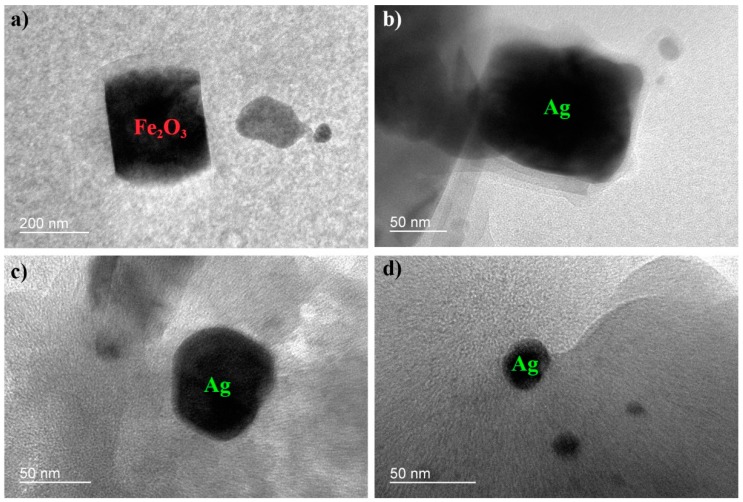
TEM images of Ag–Fe_2_O_3_@CA; (**a**): Fe_2_O_3_ nanoparticle; (**b**,**c,d**): Ag nanoparticles of various sizes.

Figure 6a,b are the bright field illumination of Ag–Fe_2_O_3_@CA. The major black particles can be assigned to Ag or Fe_2_O_3_ according to the morphology identification in Figure 5.

**Figure 7:**
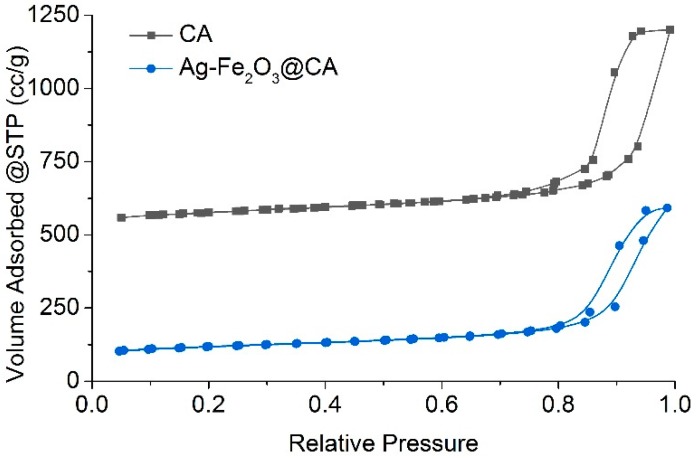
The N_2_ adsorption and desorption isotherms.

The N_2_ adsorption and desorption performances of CA and Ag–Fe_2_O_3_@CA are illustrated in Figure 7. Both of the two materials exerted IV isotherm patterns, which showed an obvious hysteresis loop at higher relative pressure. This suggested mesoporosity, as well as macroporosity, existed in both of these two materials [33]. This could be ascribed to the fact that the hosting material CA contained networks which generated from the agglomeration of nano-sized carbon particles (see Figure 5b).

In addition, the BET surface area and total pore volume of CA were found to be drastically decreased after it had hosted Ag–Fe_2_O_3_ nanoparticles (Table 1). The specific area was decreased by around 210 m^2^/g. Approximately 20% of total pore volume sacrificed owning to the growth of Ag–Fe_2_O_3_ particles on CA during the hydrothermal reaction.

**Figure 8:**
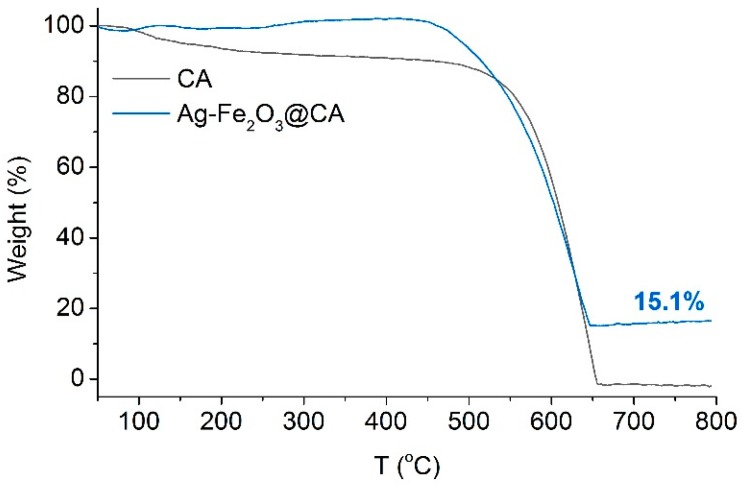
Thermal gravimetric curve of CA and Ag–Fe_2_O_3_@CA.

TGA in air gas was made to roughly quantify the content of Ag–Fe_2_O_3_ in Ag–Fe_2_O_3_@CA. According to Figure 8, it can be perceived that the weight of CA began to fall when the temperature was heated up over 100 °C, which may be due to the volatilization of unreacted residual reactants in CA substrate. The same phenomenon was not perceived in the weight change of Ag–Fe_2_O_3_@CA. This could be explained as those unreacted residual volatile reactants presented in raw CA material either having desorbed from CA or decomposed during the hydrothermal reaction. When the temperature was further heated, the weight was declined greatly because of the oxidation of solid carbon substrate forming gaseous CO_2_. Subsequently, the weight levelled off and sustained constantly at about 15.1%, which could be regarded as the approximate proportion of Ag–Fe_2_O_3_ in Ag–Fe_2_O_3_@CA.

### 3.2. Catalytic Degradation of Residual Dyes in Waste Rinsing Effluents

The catalytic performance is the most important criteria to grade the catalyst in chemical reaction. With the intention to examine the performance of catalyst materials in promoting dye degradation in effluents, both color and COD removal percentages were evaluated and compared. The probe effluent sample used was Effluent 1, which was collected from the residual baths from Rinsing 1 (see Figure 3).

#### 3.2.1. Decolorization of the First Rinsing Effluent (Effluent 1)

Decolorization of effluents is important in the recycling of waste effluent. Effective cleaning of fabrics may be impossible if completed color removal in recycled effluents was not achieved. This is because the observed color suggested there were substantial dyes presented in the rinsing bath. The wash-off step is a rinsing process, in which unfixed dyes diffused into the rinsing bath from the fabric gradually. In the event that the rinsing bath contained substantial residual dyes, the molecule diffusion of unfixed dyes to the aqueous solution would be hindered by the relatively high concentration of dyes in the rinsing bath. It was incapable of further stripping unfixed dyes on fabrics effectively by the dye-rich effluents. Consequently, residual dyes in the waste effluent had an adverse impact on the stripping of loose dyes on fabrics, during recycling of waste effluents to replace freshwater as the rinsing medium. They had to be removed prior to the recycling of waste effluents in the wash-off step, otherwise colorfastness of fabrics would be downgraded.

**Figure 9:**
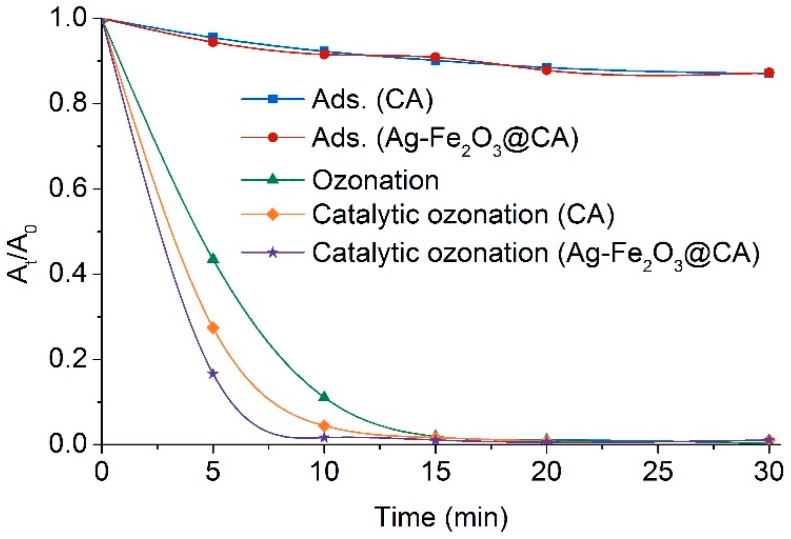
Color of Effluent 1 treated by various processes.

Color removal of effluents by ozonation, with or without catalysts, can be seen in Figure 9. In cases involving ozonation, color absorbance suddenly dropped with time in the first 15 min. Comparing these three processes, color removal in the individual case was different. Specifically, color removal efficiency increased following the order of ozonation alone, catalytic ozonation with CA, and catalytic ozonation with Ag–Fe_2_O_3_@CA. This suggested catalytic ozonation was more powerful than ozonation alone in decolorization, due to the presence of catalysts promoting dye degradation. In addition, it also could be inferred that Ag–Fe_2_O_3_@CA showed better catalytic activity in color removal comparing with CA. The most probable reason was the participation of bimetallic hybrid facilitated production of ·OH, which was responsible for much more rapid oxidation than that induced by O_3_.

According to Figure 9, close attention has to paid to the adsorption property of the catalyst materials. Previously, it has been understood that porous catalysts would remarkably strengthen color removal by physical adoption [34,35]. Notwithstanding, in the two reference processes (denoted as Ads. (CA) and Ads. (Ag–Fe_2_O_3_@CA)) which were only provided with O_2_ rather than O_3_, no more than 10% color reduction was detected in the first 15 min. The possible reason might be that the colorant in the commercial dye product has a relatively larger size than that of mesopores presented in CA or Ag–Fe_2_O_3_@CA. Another possibility was that, within the first 15 min, or even the total 30 min, neither CA nor Ag–Fe_2_O_3_@CA had fully played their adsorption capability. These reasons also could explain that even though CA and Ag–Fe_2_O_3_@CA possessed distinct porosities, they had quite identical poor adsorption capacity to the probe dyes in effluents. Since as high as 28% of the initial color reduced in the first several minutes during the treatment, it can be assumed that the improved efficiency of color removal predominantly arose from chemical oxidation promoted by catalysts rather than direct physical adsorption.

**Figure 10:**
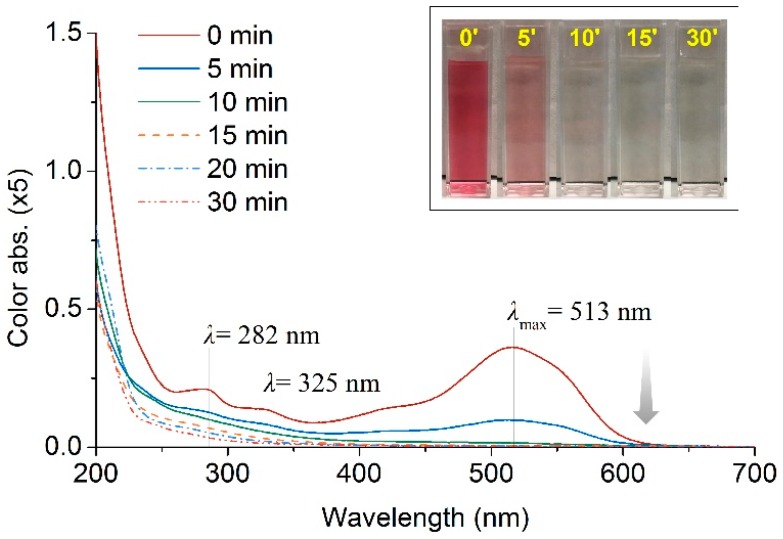
UV-vis spectrum of Effluent 1 treated by catalytic ozonation with CA.

The degradation process of residual dyes was monitored by full range UV-vis spectrum of the effluent during catalytic ozonation with CA. As can be found in Figure 10, in the visible region ranging from 400–700 nm, the strongest and broadest peak at 513 nm can be assigned to the conjugated π system stemming from the azo group (–N=N–); while the two weaker and narrower peaks located at 282 nm and 325 nm were contributed by triazine and naphthalene rings [36]. After catalytic ozonation for only 5 min, the absorbance peak at 512 nm weakened tangibly, implying the –N=N– group which acts as the chromophore system in dye molecules were substantially destructed. At the same time, the absorbance peaks from triazine and naphthalene rings at 282 nm and 325 nm were almost disappeared, suggesting naphthalene and triazine rings were also destructed significantly.

#### 3.2.2. COD Removal in the First Rinsing Effluent (Effluent 1)

Color removal is only one of the purposes in catalytic ozonation before effluent recycling. It is also necessary to understand the capacity of catalytic ozonation in degrading residual dyes approaching total mineralization, in which organics are degraded into inorganic minerals completely. Because COD quantitatively assesses the overall organic substances that can be oxidized chemically into inorganics in solution [37], it was used to precisely measure the content of organic contaminants in effluents, which potentially contained hydrolyzed completed dyes and by-products from dye degradation. With the intention of estimating the performance of catalyst materials in facilitating degradation of dye contaminants, COD removal in difference ozonation processes was evaluated and compared.

**Figure 11:**
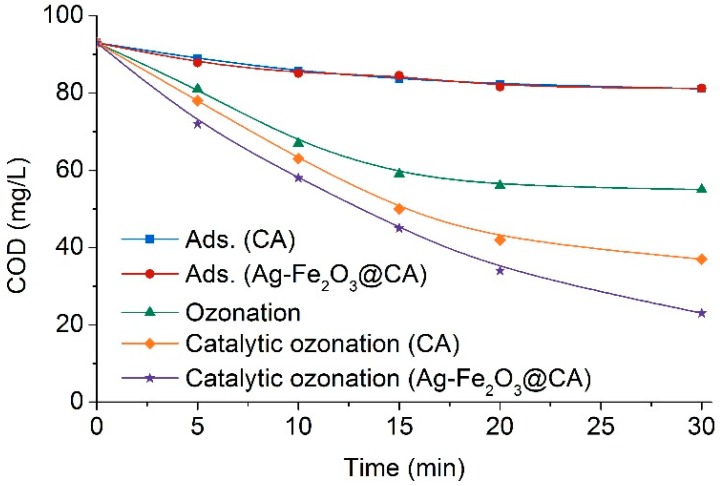
COD of the Effluent 1 treated by various processes.

Distinct to decolorization in Figure 9, COD removal in Figure 11 was much slower. COD fell with treatment time only gradually, providing compelling evidence that COD removal is much more difficult than color removal in aqueous solution. This could be explained by the fact that the effective matters influencing color and COD are totally different. For the observed effluent color, the only contributor is the dyes having a completed chromophore structure. Thus, total color removal only required primary oxidation to cleave chromophores in each individual dye. However, the contributors of COD are not only the dyes having completed chromophores, but also other organic contaminants. Thus extensive COD reduction demanded advanced oxidation of organics approaching mineralization, rather than simple cleavage of dye chromophores. As a result, the completed removal of color may rarely account for COD reduction, especially when the dye having a complicated molecular structure and high molecular weight.

In addition, as evident from Figure 11, it was also found that catalyst CA radically accelerated COD reduction. In the ozonation-alone process, COD at the end of the 30 min treatment was as high as 55 mg/L, while it was only 37 mg/L in the treatment with CA. The removal percentage had been increased from 40% to 60%. Similar to color removal, COD reduction was somewhat directly contributed by physical adsorption of dyes by CA, yielding around 13% COD reduction after half an hour. Since catalytic ozonation with CA enhanced the removal percentage by only 20% of total COD, it implied chemical that degradation of dyes contributing to COD removal did not prevail. The mostly recognized reason for this slight improvement in COD reduction, besides direct physical adsorption, was that the presence of carbon material activated ozone to decompose into ·OH [38], which displays higher oxidation capacity than O_3_ itself.

In addition, it was also observed that dye degradation in catalytic ozonation with Ag–Fe_2_O_3_@CA was much more remarkable than that with CA. COD removal percentage rose by about 30% compared to ozonation alone, and the final COD of the effluent was as minimal as 23 mg/L. As little difference of adsorption between Ag–Fe_2_O_3_@CA and CA was found, the substantial growth in COD removal percentage can be a consequence of chemical oxidation, which was dominantly induced by the catalyst Ag–Fe_2_O_3_@CA. This could be on account of that the multivalence oxidation states of Ag–Fe_2_O_3_ on CA facilitated interfacial transfer of electrons, which had been proved to play indispensable roles in oxidation [39,40].

Apart from the above, there may be another possibility that accounted for the enhanced dye degradation in the presence of catalysts. In previous studies, porous carbon materials had displayed advantageously in promoting dye removal by chemical degradation, in addition to direct physical adsorption [41]. The porous carbon may increase mass transfer of O_3_, as O_3_ may concentrate over the surface/interface of catalysts. The concentration of accumulated O_3_ tended to be much higher than that of O_3_ dissolved in the bulk solution. In addition to O_3_, dyes or its halfway oxidation by-products may also concentrate over the porous catalyst. Thus promoted chemical degradation of organics could be ignited owning to enhanced concentration of available reactants over catalyst surface/interface [42].

#### 3.2.3. Evolution of Water Quality of Effluents During Recycling

**Figure 12:**
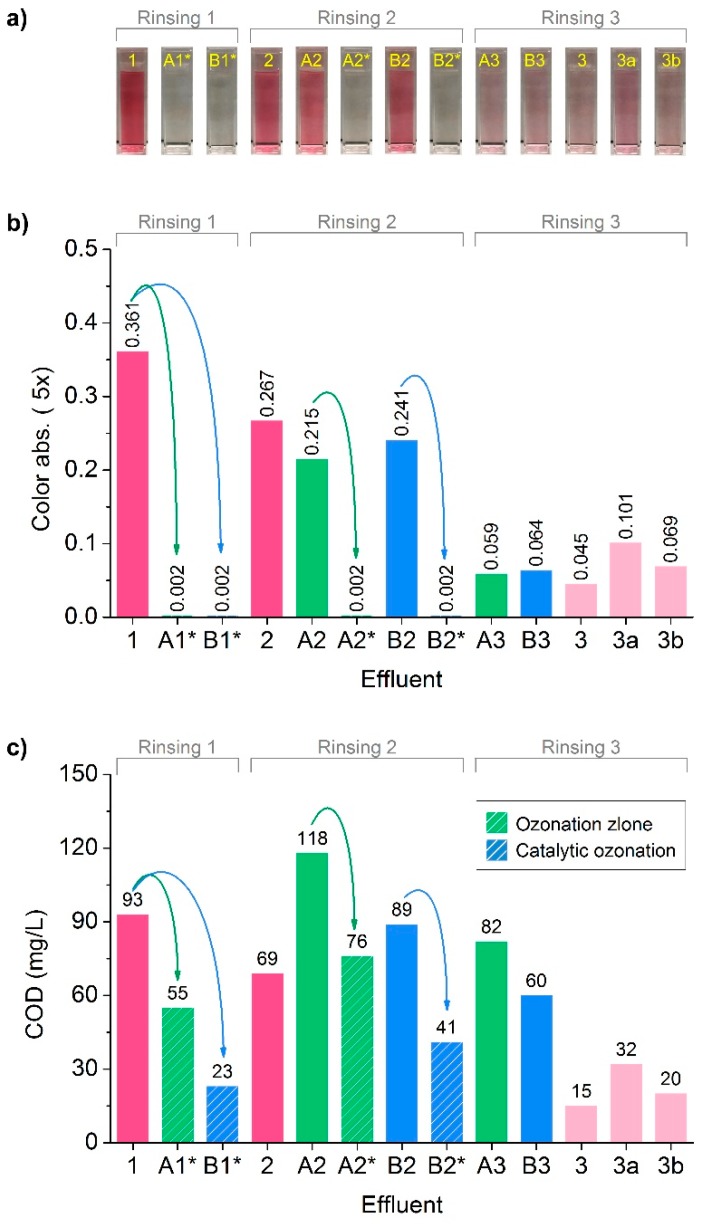
Selected effluents (**a**) and their color (**b**) and COD (**c**) evolution.

Catalytic ozonation of Effluent 1 was the first reclamation step for recycling of the waste effluents. Next, the reclaimed effluents will be reused as the rinsing medium to clean fabrics, followed by catalytic ozonation treatment again to degrade the new stripped dyes from the latest rinsing process. This reclamation–rinsing process cycle will be repeated twice to complete the total 3 rinsing processes in the wash-off step. The fate of effluents and the related fabrics has been illustrated in detail in Figure 3. During the entire recycling process, water quality of selected effluents was evaluated by color and COD. Effluent samples from three rinsing processes (Rinsing 1, Rinsing 2 and Rinsing 3) after 5 times dilution were arranged in sequence from the left to the right in Figure 12a, and the corresponding color absorbance and COD were demonstrated in Figure 12b,c.

After Rinsing 1, the resulted Effluent 1 had the highest color absorbance among all of the effluents collected in the wash-off step, according to Figure 12b. This was because Rinsing 1, which used freshwater, could clean the majority of loose dyes on fabrics right after dye fixation. After 30 min treatment with either ozonation alone or catalytic ozonation, the resulted Effluent A1* and Effluent B1* turned totally transparent without any visual color (Figure 12a), and the absorbance declined to zero (Figure 12b). After Rinsing 2, it was found that Effluent 2 showed the highest shade of color among effluents from Rinsing 2, suggesting that the recycled effluents after reclamation were not as efficient as freshwater in cleaning fabrics. This suggested that even though Effluent A1* and Effluent B1* were transparent without any color, the residual colorless contaminants presented still should be paid close attention. In addition, although the color absorbance of Effluent B2 was slightly lower than Effluent 2, it was higher than that of Effluent A2. This indicated that Effluent B1* was much closer to freshwater than Effluent A1*, in the point view of cleaning capacity. This could be attributed to the fact that catalytic ozonation was more efficient than ozonation alone in reclamation of waste effluents, because COD in Effluent B1* (23 mg/L) was much lower than that in Effluent A1* (55 mg/L) according to Figure 12c. This was also the reason that why effluents just after total color removal (around 10–15 min treatment according to Figure 9) was not adopted for recycling in the wash-off step, as they were far away from adequate reclamation for reliable reuse. After related treatments, Effluent A2 and Effluent B2 were converted to Effluent A2* and Effluent B2*, which were totally decolorized again and will be reused in Rinsing 3. After Rinsing 3, the color absorbance of Effluent A3 and Effluent B3 were similar, implying the loose dyes in the individual rinsing effluents were almost the same. Since Effluent B2 had a deeper color than Effluent A2, it could be assumed that there were still a certain amount of loose dyes presented on F1-r3. Moreover, it was also worth noting that Effluent 3b had a very limited higher color absorbance than Effluent B3. This may lead to a conclusion that, for F3 and F4, loose dyes were stripped effectively by Effluent B2* in Rinsing 2, while Rinsing 3 only stripped the minimal free dyes re-attached on fabrics. Nevertheless, color absorbance of Effluent 3a was found to be significantly higher than Effluent A3, suggesting F1 and F2 cleaned with Effluent A2* after Rinsing 2 was not adequate, as some loose dyes was further stripped from F2-r2 using freshwater after Rinsing 3.

As can be seen in Figure 12c, different COD was presented in Effluent A2^*^ (76 mg/L) and Effluent B2^*^ (41 mg/L). However, the difference hardly influenced their cleaning capacity in Rinsing 3, as color absorbance of Effluent A3 and Effluent B3 were almost the same according to Figure 12b. This was quite different from that in Rinsing 2, where the difference in color absorbance of Effluent A2 and Effluent B2 was noticeable, indicating the cleaning capacity of Effluent A1* and Effluent B1* was impacted remarkably by their residual COD. This might be ascribed to the different contents of loose dyes on fabrics. Before Rinsing 2, fabrics still contained appreciable loose dyes. The substantivity of dyes towards fabrics was likely to be increased by those oxidation by-products from ozonation in effluents, leading to decreased cleaning efficiency of the recycled effluents. By contrast, after Rinsing 2, there was only few dyes on fabrics. Thus, oxidation by-products which contributed to COD hardly decrease cleaning capacity of the recycled effluents. This assumption could be supported by the fact that Effluent 3 displayed the minimal color comparing to Effluent 1 and Effluent 2. In addition, COD in effluents accumulated in different effluents even though after treatments by ozonation alone or catalytic ozonation. This was different from color, which was calibrated to zero after treatments. As discussed previously, this phenomenon derived from the deduction that COD removal was more difficult than color. Thus, total color removal could not result in total COD removal. The oxidation by-products from ozonation treatments would be mixed with newly stripped dyes from fabrics, leading to increasingly accumulated COD. Nevertheless, owning to efficient ozonation treatments, the final COD of both Effluent A3 (82 mg/L) and Effluent B3 (60 mg/L) was no more than that of Effluent 1 (93 mg/L). It was conceivable that recycling effluents by of ozonation treatments not only extremely reduced water consumption, but also decrease contamination in waste effluents.

### 3.3. Color Quality of Fabrics

#### 3.3.1. Color Reproducibility

Evaluation of color and COD removal only confirms the effect of ozonation treatments on dye degradation in waste effluents. It is more important to ascertain whether fabrics could maintain its integrity in color reproduction when the rinsing medium was replaced from freshwater to recycled effluents. For the sake of color reproducibility, fabric samples cleaned with different rinsing mediums were compared in color specifications by the CIELAB color space system.

As listed in Table 2, F1-r3, F2-r3, F3-r3 and F4-r3 possessed lower L* but higher a* and b* than those of the reference fabric F5-r3. This indicated these 4 fabrics which had been rinsed with recycled effluents were slightly darker, greener and yellower in shade than F5-r3, according to the three-dimensional CIELAB color space system [43]. The variations of color parameters were mainly a consequence of the impact from the oxidation by-products from dye degradation in recycled effluents. The presence of these organics decreased the cleaning capacity of recycled effluents to strip loose dyes from fabrics.

*ΔE_cmc(2:1)_* is the most critical color specification measuring color difference between 2 different fabrics objectively. The smaller the *ΔE_cmc(2:1)_*, the better the color reproducibility. As is observed in Table 2, *ΔE_cmc(2:1)_* of F1-r3, F2-r3, F3-r3 and F4-r3 were positive, suggesting recycled effluents were not as capable as freshwater in cleaning of fabrics for stripping loose dyes. Since the *ΔE_cmc(2:1)_* of F1-r3, F2-r3, F3-r3 and F4-r3 were not the same, this indicated that the varied rinsing recipes (see Figure 3) resulted in different deviations in color reproducibility. As smaller *ΔE_cmc(2:1)_* indicates it being more difficult to tell the minimal color difference by the human naked eye, it can be speculated that F3-r3 and F4-r3 were much closer to F5-r3 in color, than F1-r3 and F2-r3. This could be ascribed to the fact that, according to Figure 12b, Effluent B1* was more capable than Effluent A1* in cleaning of fabrics in Rinsing 2. As Effluent B1* and Effluent A1* were reclaimed by catalytic ozonation and ozonation alone respectively, it could be inferred that catalytic ozonation with Ag–Fe_2_O_3_@CA had tangibly eliminated organic by-products from dye degradation in effluents.

In addition, when closely comparing F3 with F4 and F1 with F2, it was found that F1 and F3 were of poorer color reproducibility than F2 and F4, respectively. It could be inferred that freshwater was essentially advantageous in cleaning of loose dyes in Rinsing 3. However, in Rinsing 3, freshwater was not capable enough to thoroughly strip loose dyes which should be stripped in Rinsing 2, as *ΔE_cmc(2:1)_* of F2-r3 was much higher than that of F4-r3 by more than 1.5 unit. This could be explained that Rinsing 2 performed at 95 °C was more efficient than Rinsing 3 in stripping of loose dyes. Even though freshwater was used in Rinsing 3, it was still incapable of covering the shortage of inadequate cleaning of loose dyes in Rinsing 2. This was in accordance with the conclusion made according to Figure 12b that Rinsing 3 only stripped the minimal free dyes re-attached on fabrics, but rarely striped those loose dyes which should be stripped in Rinsing 2 at high temperature only.

In general, the tolerance in color reproducibility is *ΔE_cmc(2:1)_* < 1.0, as within this deviation the human naked eye cannot tell the color difference between two different fabrics [13]. Thereby, it was conceivable that F3-r3 and F4-r3 displayed acceptable color difference within the visible detection limit, while F1-r3 and F2-r3 were inferior from the viewpoint of color reproducibility. Consequently, it can be validated that catalytic ozonation with Ag–Fe_2_O_3_@CA had successfully reclaimed waste effluents to maintain color reproducibility of fabrics during the recycling process, in which freshwater was drastically eliminated. By contrast, ozonation alone without catalysts was found to be incapable to renovate waste effluents for reuse in rinsing, as color similarity of fabrics was practically inferior and more importantly was out of the acceptable limit.

#### 3.3.2. Colorfastness

As mentioned earlier, the wash-off step in entire reactive dyeing is a cleaning process for improvement of colorfastness of fabrics after dye fixation. Thus, colorfastness is another ultimate consideration in the wash-off step. Colorfastness of fabrics is listed and compared Table 3, in which the fabric (F0) without wash-off was also included.

As can be observed in Table 3, F0 exerted the worst colorfastness. It was around 1–2 grades lower than the reference F5-r3, supporting the conclusion that fabrics without the wash-off step were seriously downgraded in colorfastness. Colorfastness of F1-r3 and F2-r3 were about 0.5–1 grade lower than those of F5-r3, suggesting the related wash-off step was not sufficiently stripped the loose dyes to meet up with universal criteria for textile colorfastness. However, F3-r3 and F4-r3 were of equivalent colorfastness comparing to F5-r3. Thus, it was convincing that catalytic ozonation in the presence of Ag–Fe_2_O_3_@CA was a feasible technique in reclamation of waste rinsing effluents for recycling. The wash-off efficiency of recycled effluents was almost as same as that of freshwater.

## 4. Conclusions

Recycling of wastewater is getting increasing environmental concern in textile processing. Very little information is available on the minimization of freshwater consumption in the wash-off step in reactive dyeing. In the present study, the catalyst Ag–Fe_2_O_3_@CA has been adopted in catalytic ozonation for reclamation of waste rinsing effluents, pursuing effluent recycling to reduce freshwater consumption. It was found that the participation of the catalyst significantly facilitated dye degradation in catalytic ozonation of waste effluents. The reclaimed effluents can be reused repeatedly as rinsing baths to strip loose dyes attached on fabrics, which were rarely downgraded in both color reproducibility and colorfastness. By contrast, ozonation alone without the catalyst in the same conditions could not secure the final color quality of fabrics, of which color difference was out of the tolerance and colorfastness did not meet with acceptable criteria. To our knowledge, this is the earliest evidence provided to demonstrate the potential of catalytic ozonation in minimizing water consumption in the wash-off step in textile reactive dyeing. It may bridge gaps in sustainable development in the textile industry, which would benefit both the industry and the community.

## Figures and Tables

**Table 1:** Textural property of CA and Ag–Fe_2_O_3_@CA.

Materials	BET (m^2^/g)	Pore radius (nm)	Pore volume (cc/g)
CA	586.7	8.77	1.07
Ag–Fe_2_O_3_@CA	378.6	9.07	0.81

**Table 2:** Color specifications of fabrics rinsed with freshwater or treated effluents.

Fabric	L*	a*	b*	*ΔL*	*Δa*	*Δb*	*ΔE_cmc(2:1)_*
F5-r3 (ref.)	30.66	45.40	9.27	-	-	-	-
F1-r3	27.80	46.93	11.59	−2.86	1.53	2.32	2.37
F2-r3	28.12	46.59	10.72	−2.54	1.19	1.45	1.90
F3-r3	30.19	45.71	9.76	−0.47	0.31	0.49	0.43
F4-r3	30.13	45.66	9.39	−0.53	0.26	0.12	0.35

**Table 3:** Colorfastness of fabrics rinsed with freshwater and treated effluents.

Fabric Samples	Laundering		Crocking	
	Staining on Cotton	Color Change	Dry	Wet
F5-r3 (ref.)	4.5	5	5	4.5
F0	3.5	3	4	3
F1-r3	3	3.5	4	3.5
F2-r3	4	4	4.5	4
F3-r3	4.5	4.5	4.5	4.5
F4-r3	4.5	5	5	4.5
